# Quality of life data from EQ-5D for evidence-based health service practice in dialysis care

**DOI:** 10.1186/1472-6963-14-S2-P37

**Published:** 2014-07-07

**Authors:** Klaus Eichler, Sascha Hess, Mathias Früh, Oliver Reich, Urs Brügger

**Affiliations:** 1 Winterthur Institute of Health Economics, Zurich University of Applied Sciences, 8401 Winterthur, Switzerland; 2 Department for Health Sciences, Helsana Insurances AG, 8081 Zurich, Switzerland

## Background

Hemodialysis (HD) and peritoneal dialysis (PD) are therapeutic options for patients with end-stage-renal-disease (ESRD), if transplantation is not available. Mortality rates for HD and PD are similar, while PD is generally the less costly alternative. Percentage of HD and PD shows considerable variability between high income countries (for PD from 5-7% in Germany and Switzerland up to 19-24% in the UK and Scandinavia). Patient reported outcomes, such as quality of life (QOL), can provide complementary evidence for planning of patient oriented dialysis services. Profile instruments (e.g. SF36, KDQOL) show no consistent QOL differences between HD and PD. However, single index preference-based QOL measures (such as EQ-5D), may add new information and are useful for later health economic evaluations. We aimed to collect current evidence for QOL of ESRD patients as measured with EQ-5D.

## Materials and methods

We performed a systematic literature search in electronic databases (Medline; Cochrane Library; from 2000 to March 2014; no language restriction). In addition, data from registries and manufacturers were included. We included experimental (RCT) and observational (cohort, cross-sectional) studies, which compared QOL between HD and PD-patients with EQ-5D in high income countries. Two reviewers screened titles and abstracts, resolved disagreements and extracted data. QOL data were pooled with two separate metaanalyses: (1) EQ-5D-VAS values as “patient view valuation” and (2) EQ-5D-index values as “general population view valuation”.

## Results

We retrieved 962 references. Six studies (with 8 comparisons), mostly from routine care, fulfilled inclusion criteria. The pooled difference in QOL between HD and PD, as derived with EQ-5D visual-analog-scale (VAS: 0 to 100), was 1.4 (95%-CI: -2.0 to 4.8) in favor of PD. The difference in QOL, as derived with the EQ-5D index values (0 to 1), was 0.08 (95%-CI: -0.05 to 0.22), again in favor of PD. These differences, however, may not be clinically relevant nor are they statistically significant. In addition, substantial heterogeneity emerged for the EQ-5D index value analysis (12:89.7%).

## Conclusions

QOL as measured with the single index preference-based instrument EQ-5D was similar for HD and PD, but data is scarce. More real world QOL data are needed from Health Services Research to increase precision of results. This may also improve knowledge about minimal important differences in QOL for ESRD patients as measured with EQ-5D. Finally, QOL data from EQ-5D could contribute patient oriented evidence for design of health services with an optimal balance between HD and PD care.

